# Surgery That Is Surgery

**Published:** 1871-07

**Authors:** 


### SURGERY THAT IS SURGERY.

In the June number of the Dental *Cosmos*, page 313, a dentist is represented as saying he "extracts the first molars when the second come in;" but we are not told whether he extracts the second, when the third make their appearance, or whether he extracts the central incisors when the lateral "come in." It is a pity we have not a full report on all these things ; but probably space would not permit.

Now, it is not strange, that a man who, in the name of science, mutilates the human body, which our Father in Heaven has so "fearfully and wonderfully made," speaks "of the defects of approximal fillings at the base, and considered it" (what?) "as probably due to soft foil. In such cases, cuts out the softened portions, and fills with amalgam, leaving the firm part of the gold standing: thought not the slightest harm could come from putting gold and amalgam in contact."

It is wicked to swear, and impolite to scold; and so we can not do justice to the subject. Of the man who holds such sentiments we have nothing to say. We don't know him. Of the sentiments, it may be said, that if our profession can rise with such dead weights attached, it has a most marvelous force of character. W.

